# Dr. Adams, in Answer to Mr. Young

**Published:** 1806-08

**Authors:** Joseph Adams

**Affiliations:** Berner's Street


Medical and Phyfical Journal.

VOL. XVI.] AUGUST, 1806. [NO. 90.

*Printed for R. PHILLIPS, by W. Thorne, Red Lion Court, Fleet Street, London.*

### To Mr. YOUNG.

SIR,

NOTHING but my close engagements, in bringing a new edition of "MORBID POISONS" before the public, should have induced me so long to disregard your letter in the 87th number of the Journal; because nothing is so agreeable to me as those questions, which may render a theory better understood, or, by proving its fallacy, engage the framer in a new set of inquiries.

Let me first observe, that I have never yet entered into the question, whether hydatids found in the liver have a life and economy independent of the animal in which they are found, excepting as they draw their nourishment from it: Satisfied of the truth of the position, having no claim to the discovery, nor any new arguments to offer in its support, it seemed more decent to transcribe the words of those to whose labour we are indebted. It does not indeed make a necessary part of my theory, which only goes to show, that there is the same reason to admit a separate existence in one species of cancerous breast as in the hydatids of the liver; and that as the human mamma is found a convenient nidus for the common hydatid, there is a presumption that other substances found in it, may have a similar economy.

The only proof we require of life is motion, independent of elasticity. In the common adipose cellular membrane, we have nothing like motion. There is, indeed, some elasticity in the fibres, but this is different from the motion of life, because it exists after death with the same force. The question at issue therefore is, whether the deposition of fat into regular cells, laterally attached to, yet, like the cells of a honey comb, having no communication with each other, which cells show a contractile power by the elevation of their contents, yet, which cannot be the effect of elasticity, because it ceases with life:—and whether such a process as this is to be imputed to the growth and multiplication of an animal like the hydatid, or whether it is to be considered as the mere adhesion of the sides of cells by the effusion of lymph? The effusion of lymph might consolidate the whole substance, as we find it obliterate the air cells of the lungs; but this is very different from the formation of regular and uniform cells. You observe very justly, that the sides of abscesses are thickened. But we see no muscular contraction in these sides, nor do we ever see a series of abscesses resembling each other in size and form, attached laterally to each other, and filled with a secretion which is found in every part of a healthy body; but in this particular part, increased in proportion as the disease advances.

You ask, whether it is possible to trace the line of distinction between the diseased fat, as it is called, or, as I term it, carcinomatous hydatids, and the healthy fat? Nothing Sir, is so easy; for it is impossible to mistake them. The colour of the diseased fat, or of the contents of carcinomatous hydatids, remains longer after exposure, the regularity of the papillæ is not less striking, and the facility with which each cell is emptied of fat, unconnected with fibres, make a distinction which can never be mistaken. But I could wish it to be remembered, that it is only one species of cancerous breast, which I call carcinoma, that exhibits this appearance. You will also recollect, that I have never met with this appearance excepting in cancer, seated in the breast or lips; and even in these parts the variety of cancers is so great, that my principal object was, to induce surgeons, by constantly examining the contents of amputated lumps, and recording the issue of their operations, to ascertain the probability of success in all future cases.

This last consideration will oblige me to be much shorter in my reply, than the long letter with which you have honoured me. It is unnecessary to dwell on your objection, that the hydatis lymphatica is propagated within the parent, by the bursting of which, the young ones escape, and that no such process can take place in cysts attached laterally to each other. A moment's recollection will remind you, that among the varieties of hydatis lymphatica, many are multiplied merely from the sides of each other. The same objection occurs to your observation, that some of of these carcinomatous hydatids are at a distance from each other, and separated by the natural structure of the breast. The same is found in the common hydatids of the liver.*

It is very true, that the constant sloughing of the cyst of an hydatid, or of a melicerous or steatomatous tumour, and their total incapacity, after being partially amputated, to form a healthy adhesion with the neighbouring parts, only marks an *uniform difference* between the cysts of these substances and the capsules of abscesses, or the tunics of original formed parts. It is not less true, that the uniformity of that difference is not a sufficient proof of the animalcular nature of these cysts: nor have I considered it as such; but it is at least a presumptive proof, that substances which preserve so constant a law, uniformly different from other substances subjected to the same operation, should have properties common to each other, and different from those whose actions are uniformly governed by a different law. That is, if the cysts of hydatids, or steatomatous and melicerous tumours, never can be made to granulate; if the capsules of abscesses always granulate, and the tunics of original formed parts when divided, either adhere or granulate, there must be some laws which uniformly govern each, and which by this uniformity, argue a similarity in the properties of those which are governed by similar laws.

When you speak of the cancerous wart, you remind me of another gentleman, I believe Mr. Burns, who makes the same objection to my theory. When Mr. Burns wrote, I conceived I might not have sufficiently explained myself; but as, since then, I have devoted a book to that subject alone, and in order to render myself more intelligible, to correct my ideas if faulty, to vary my mode of expression according to the ideas of others, and to bring every view of the question before the public, I have been indulged with the correspondence of the ablest men in the metropolis, and allowed to publish our correspondence:—after this, if I am not intelligible, I must despair of being so, and trust to the industry of others in recording what they discover, or to future opportunities of demonstrating in the recent subject what I find it in vain to describe in words.

* See my letter to Mr. Abernethy in the published Correspondence.

 I am

I am however, Sir, not the less obliged to you for your letter. The oftener the subject is brought before the public, the greater will the probability be, of hereafter finding if not a remedy, at least an alleviation of that dreadful disease, peculiarly fatal to a sex which is debarred the means of inquiry, and calling for our sympathy, not less on account of its fatality than the misery which attends every stage of its progress.

I am, SIR, &c.

*Berner's Street, July 5, 1806.* JOSEPH ADAMS.

